# Short term effects of a novel combined approach compared with physical therapy alone among older patients with temporomandibular degenerative joint disease: a prospective cohort study

**DOI:** 10.1186/s12903-023-02848-9

**Published:** 2023-03-25

**Authors:** Shasha Liu, Shuai Fan, Guiping Li, Bin Cai, Yuan Yao, Lei Jin, Yuxin Zhang, Xinjun Zhang, Lili Xu

**Affiliations:** 1 Department of Rehabilitation Medicine, The Ninth People’s Hospital, Shanghai Jiao Tong University School of Medicine, Shanghai, China; 2 Department of Rehabilitation Medicine, Sijing Hospital of the Songjiang District of Shanghai, Shanghai, China; 3 Department of Orthopedics, Sijing Hospital of the Songjiang District of Shanghai, Shanghai, China

**Keywords:** Temporomandibular Joint Disorders, Middle aged, Aged, Osteoarthritis, Injections, Exercise, Physical therapy modalities

## Abstract

**Background:**

There is a lack of consensus regarding the best treatment option, including physical exercise, available for temporomandibular degenerative joint disease (DJD) that affect the older patients. Herein, we aimed to study and compare the efficacy of a combined approach using injection and home physical exercise with physical therapy alone as well as explored an optimal treatment strategy for older patients with DJD.

**Methods:**

We included 213 older patients with DJD treated at our medical centre from June 2020 to June 2021, 64 of whom were selected for analysis. Of these 64 patients, 32 received injections combined with home physical exercise, and the other 32 received physical therapy alone. Propensity score matching was used to ensure that the two groups did not differ significantly in categorical and continuous variables. Measurements included pain intensity, maximum mouth opening, joint crepitus, jaw functional limitation scale (JFLS) scores, treatment times, and treatment durations. Improvement in each measurement was compared between the two groups 2, 4, and 12 weeks after the treatment commenced, as were the final treatment times and durations.

**Results:**

Pain intensity, maximum mouth opening, and JFLS scores in the two groups improved 2, 4, and 12 weeks after treatment (all p < 0.05). The crepitus ratio improved significantly only in the combined treatment group after 12 weeks. Compared with the physical therapy group, pain while opening the mouth improved after 2, 4, and 12 weeks in the combined treatment group. Furthermore, 2 weeks after treatment, the physical therapy group showed significant improvement in maximal mouth opening compared with the combined treatment group. No significant between-group differences were observed regarding improvement in joint crepitus and JFLS scores at each observation point. The combined treatment group had a significantly lower number of visits than the physical therapy group; however, the treatment duration was longer.

**Conclusion:**

Compared with physical therapy, pain while opening the mouth is significantly improved by the combined treatment within 12 weeks, and the number of required visits is fewer. Physical therapy improves the patients’ mouth-opening capabilities in a short time (2 weeks), and the treatment cycle is short.

## Background

Temporomandibular disorders (TMDs) affect the masticatory muscles and temporomandibular joint (TMJ), as well as related tissues, with an incidence rate of 10–15% in the general population [1].

Degenerative joint disease (DJD) of the temporomandibular joint, also known as TMJ osteoarthritis, is an important TMD subtype. Its main clinical symptoms include pain, crepitus, and limited joint movement [2, 3], with associated degenerative changes of the TMJ visualized through various imaging techniques [4]. DJD often affects TMJ-related functions such as chewing, speech, and facial expressions, thereby decreasing the patients’ quality of life. Degenerative changes of the TMJ are closely related to increased age, and DJD is more common in the older individuals [5, 6].

TMD treatment includes three main objectives: reducing pain, reconstructing normal mandibular movement, and improving the patients’ quality of life [7]. Effective treatments for DJD include drugs, intra-articular injections, physical therapy, and splints [7, 8]. Prosthodontics is often applied in patients with TMD, aiming to achieve patient comfort, oral stability, and the complex restoration of the tooth [9]. Furthermore, some evidence suggests that Botulinum toxin type A injection can alleviate TMD-related pain in a short term [10]. Therefore, since conservative treatment can is reversible and can effectively alleviate pain, it should be the first choice in patients with TMD [11]. Physical therapy is typically an initial treatment recommended for TMD.

It is well-established that physical therapy can relieve the pain of TMD, improve maximum mouth opening (MMO), and jaw function [12, 13]. Physical therapy usually encompasses multiple treatment methods, including interference electrotherapy, low-intensity lasers, ultrasound, other physical factor treatments, soft tissue massage technology, joint mobilization, and physical exercise [14]. Regarding TMD treatment, physical exercise can be used as part of the physical therapy or in isolation [15, 16].

In recent years, increasing attention has been paid to the relationship between head and neck posture and TMD, although the relationship remains unclear [17, 18]. As a part of individualized comprehensive physical therapy, head and neck posture training can effectively improve the maximum painless mouth opening in patients with TMD and their overall activities of daily living [19, 20].

Intra-articular injections are another common treatment of DJD that control pain symptoms effectively. The injection drugs include hyaluronic acid, corticosteroids, and platelet-rich plasma [21–23] Physical exercise should be conducted without obvious pain [24]. Hence, it is generally recommended either in combination with drugs or injection therapy, to ensure complete pain alleviation in patients [25–27].

Many clinical studies have been conducted on DJD treatment; however, few studies have been conducted on DJD treatment in the older patients. In addition, few studies have been conducted on the effects of physical exercise in patients with DJD. Hence, our study aimed to explore the efficacy of a combined approach including injections and home physical exercises compared with physical therapy alone. We further explored an optimal treatment strategy for older patients with DJD.

## Methods

This was a prospective cohort study, and the reporting of this work followed the guidelines delineated in the STROBE statement [28]. This study was registered in the Chinese Clinical Trial Registry (registration date: 28/05/2020, registration number: ChiCTR2000033328).

**Fig. 1:**
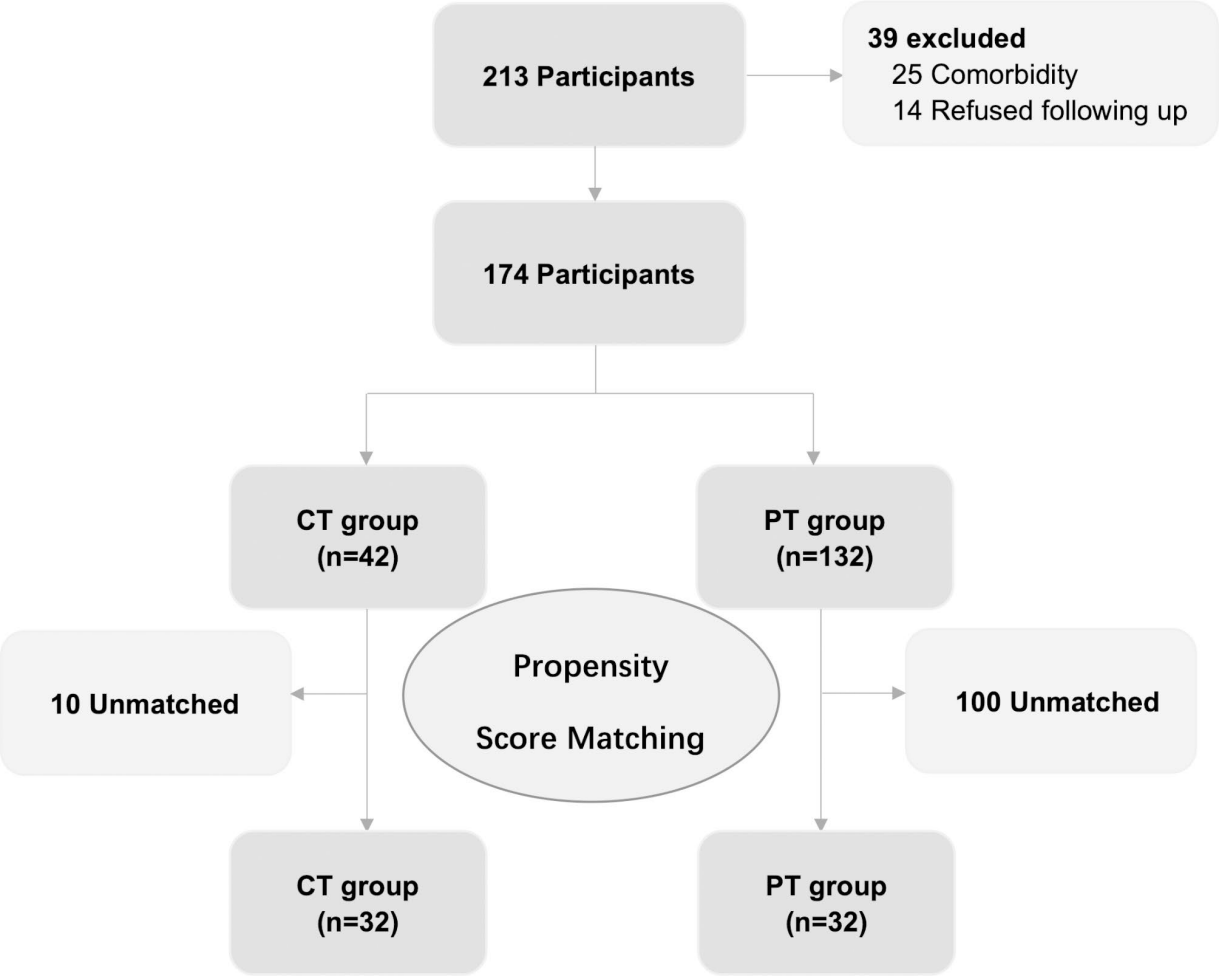
Flowchart of the sample selection process CT, combined treatment; DJD, degenerative joint disease; PT, physical therapy

Older patients with DJD who received injection therapy combined with home physical exercise or physical therapy alone in the Department of Temporomandibular Joint Rehabilitation at the Ninth People’s Hospital (affiliated with the Shanghai Jiao Tong University School of Medicine) from June 2020 to June 2021 were included in our study. Propensity score matching (PSM) was used for 1:1 nearest-neighbour matching, and appropriate cases were selected for the final statistical analysis (Fig. 1).

This study followed the principles of the Declaration of Helsinki and received approval from the ethics committee of the Ninth People’s Hospital. All patients provided their informed consent before the treatment.

### Inclusion and exclusion criteria

The inclusion criteria were as follows: pain symptoms and satisfaction of the DJD diagnostic criteria according to the Diagnostic Criteria for Temporomandibular Disorders guidelines [4]; age ≥ 50 years; magnetic resonance imaging (MRI) examination performed before enrolment; and provision of written informed consent.

The exclusion criteria were as follows: ongoing TMD treatment within 3 months before the commencement of the evaluated treatment; infections in the joint area or other conditions that are unsuitable for injection or physical therapy; history of surgery in the TMJ area; neurological diseases, immune system diseases, cognitive impairment, tumours, and other diseases and disorders considered unsuitable for inclusion in our study by the consulting physicians.

### Treatment

The specialists educated all patients in this study before treatment, explaining the anatomy and function of the TMJ and the aetiology, prognosis, and treatment plans for DJD, and correcting the patient’s daily oral behaviours (such as unilateral chewing, habitual tooth clenching, excessive eating of hard food).

#### Combined Treatment Group

Patients in the combined treatment group were treated with injection therapy and home physical exercise. The routine injection therapy involved injecting hyaluronic acid (HA) into the superior (0.6 mL) and inferior joint spaces (0.6 mL) under the guidance of ultrasound three times, with a 2-week interval between each injection.

However, if the patient had severe pain (visual analogue scale [VAS] score > 6) and MRI revealed numerous effusions in the superior joint space, a mixture containing betamethasone and lidocaine was injected into the superior joint space under ultrasound guidance (once initially, 0.5 mL dexamethasone + 0.5 mL lidocaine). Thereafter, HA was injected into the superior (0.6 mL) and inferior joint spaces (0.6 mL) under the guidance of ultrasound twice, with a 2-week interval between injections.

**Fig. 2:**
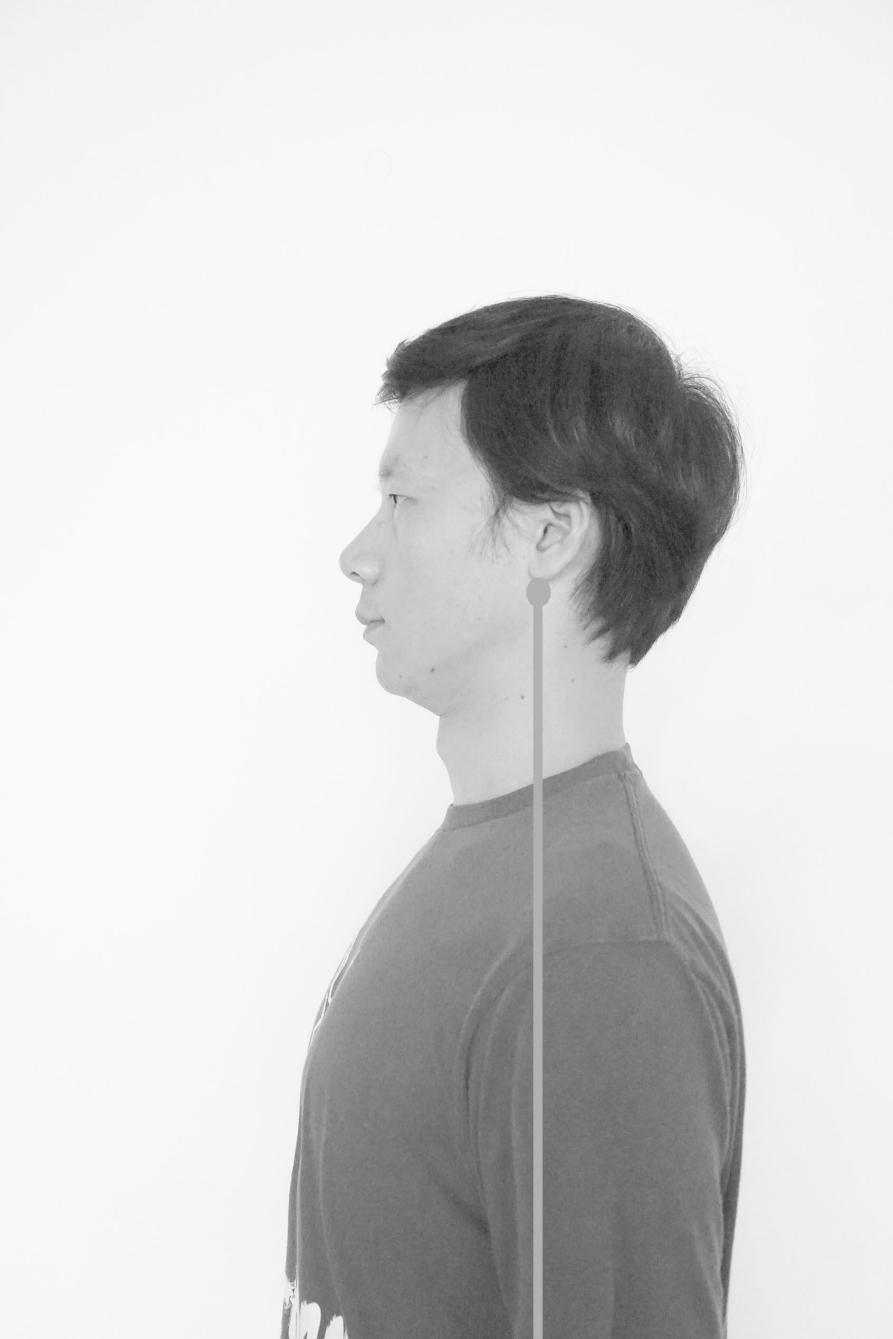
Postural exercise Maintaining correct neck and shoulder posture and resting position of the jaw

**Fig. 3:**
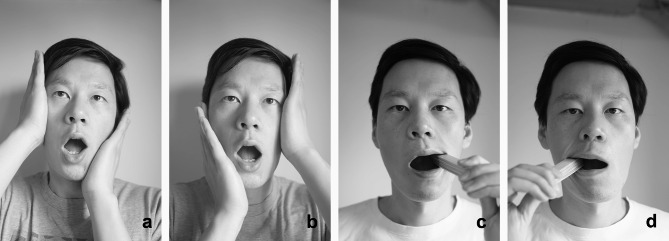
Stretching exercise a and b: mandibular body-condylar cross-pressure exercise c and d: tongue depressor exercise

**Fig. 4:**
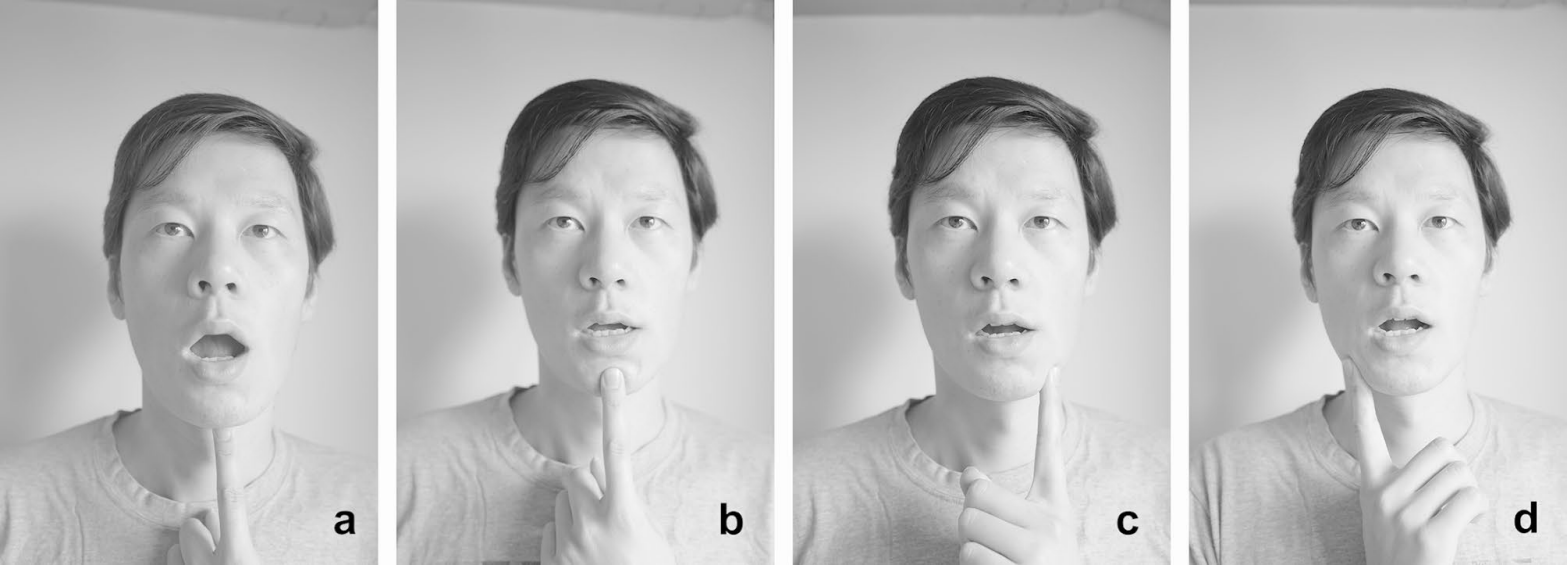
Muscle strengthening exercise (Resistance training)

**Fig. 5:**
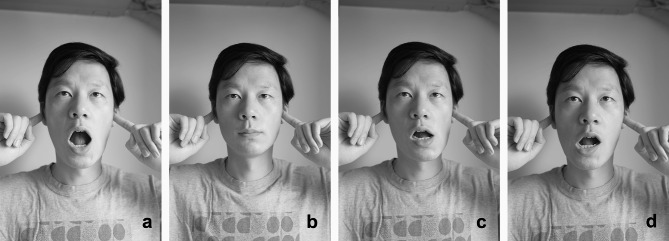
Coordination exercise Simultaneously activating agonist and antagonist masticatory muscles

In addition, home physical exercise was conducted twice daily for 12 weeks. A therapist guided patients to complete training before the first treatment and before each injection to ensure the correctness of action execution in older patients. Patients visited the hospital for separate exercise training after the third injection when the exercise prescription needed adjustment. A training manual was distributed to the patients. All patients recorded their self-training every day in a treatment diary. The home physical exercise intervention included four components: postural [19], stretching, muscle strengthening, and coordination exercise interventions [29] (see Figs. 2, 3, 4 and 5).

##### Postural Exercise

This intervention aimed to guide the patients in understanding the resting position of the jaw [30] and correcting the bad posture of the neck and shoulder. Specifically, this intervention involves maintaining the neutral position of the head, slightly retracting the jaw, relaxing the shoulders, and aligning the earlobe with the centre of gravity of the body to reduce jaw retraction caused by forward flexion of the neck. The upper and lower teeth are naturally separated by 2–4 mm, the lower jaw and masticatory muscles remain relaxed, the front one-third of the tongue is naturally attached to the rear of the upper incisor and the front portion of the upper jaw, and the lips are gently closed. Additionally, patients were instructed to adopt nasal breathing. Patients should endeavour to maintain the above positions when sitting or standing.

##### Stretching Exercise

This intervention aimed to stimulate and stretch the joint capsule and relax the masticatory muscles. In the mandibular body-condylar cross-pressure exercise, the patient presses one hand against the zygomatic bone and the other against the contralateral mandible while slowly opening and closing the jaw. This exercise can be repeated 10 times on both the sides with no or only slight pain. If the pain is aggravated on one side and relieved on the other, relative pressure should only be performed on the pain-relieved side.

In the tongue depressor exercise, the patient places the stacked tongue depressor between the upper and lower molars of the affected side, parallel to the direction of the dentition on the affected side. When conscious mouth opening can be increased during this period, the number of tongue depressors should be increased. The whole process is performed with no or only slight pain. The drafting time increases from 1 min on the first day to 5 min after 1 week.

##### Muscle Strengthening Exercise (Resistance Training)

This intervention aimed to practice the control of masticatory muscle output in terms of strength, time, and direction. Specifically, the patient places his or her index finger in four directions on the front, bottom, left, and right of the mandible to conduct strength training at the junction between the mandible and the index finger. The resistance intensity is set to 10–30% of the maximum strength in each direction of the mandible. The exercises are repeated 10 times continuously in each direction for 10 s each.

##### Coordination Exercise

This exercise aimed to improve unbalanced muscle activity by simultaneously activating agonist and antagonist muscles to maintain the appropriate mandibular movement trajectory. When practicing this exercise, the patient faces the mirror and touches the condyle with the index finger of both hands to feel the movement of the condyle and practice the mandibular movement of opening and closing the mouth and moving the left and right halves laterally. When opening and closing the mouth, the patient must ensure that the bilateral condyles slide forward and downward simultaneously at the same amplitude. The lateral movement is completed by opening the mouth with one finger. When leaning to the left, the right condyle slides slightly backward, and the left condyle slides to the front and lower left. The action can be held at the end of the maximum range for 1–2 s and should be repeated 10 times. This intervention should be practiced with no or only slight pain.

#### Physical Therapy Group

This group received physical therapy two to three times per week for 3 weeks. Patients had the option of discontinuing treatment early if there was no pain, jaw function was normal, and daily life was unaffected. However, if the patient did not feel better at the end of 3-week intervention, the treatment time was extended until the patient felt that he/she had recovered or there was no progress in the treatment. Otherwise, the treatment was terminated after 12 weeks.

Physical therapy included (a) physical factor therapy: all patients received ultrashort wave therapy [31] (45 w, 15 min), low-intensity laser [32, 33] (780 nm, 10 min), interference electrotherapy [19] (100 Hz, 15 min), and ultrasound therapy [34] (1.0 W/cm^2^, 3 min) to the affected TMJ area (ultrashort wave, ultrasound, low-intensity laser, and interference electricity); (b) manual therapy (including joint mobilization and soft tissue massage of the TMJ and cervical region [35], 30 min); and (c) exercises [19, 35] completed under the guidance of a physiotherapist at the hospital (20–30 min). The patients in the physical therapy group completed steps (a)–(b), which occurred in about 1.5 h.

### Measurements

Pain intensity, MMO, joint crepitus, and mandibular function (jaw functional limitation scale [JFLS] score) were evaluated at baseline and followed up at 2, 4, and 12 weeks after the treatment commenced.

Pain intensity was assessed using VAS (0–10 points), wherein 0 represents no pain and 10 represents the maximum intensity of orofacial pain that the patient can experience. The patients used a pain diary to record VAS scores in three states daily: chewing most of their food, actively opening their mouth to maximum capacity, and resting. The VAS score representing the patient’s severest pain was also recorded daily. MMO was measured with a millimetre ruler; this included measuring and recording the distance between the upper and lower incisors given the patient’s maximum active mouth opening (three times continuously), taking the average value, and adding or subtracting according to the coverage of the upper and lower incisors when the mouth was closed. In this procedure, the doctor palpated the patient’s bilateral TMJ area with both hands, instructed the patient to open and close the mouth, move the jaw forward and laterally three times each, and recorded the crepitus of the joint area.

Jaw function was assessed using the eight-term version of the JFLS, including eight items evaluating overall limitations in chewing, jaw movement, speech, and emotional expression. The function rating scale ranges from “no limit” (0 points) to “severe limitations” (10 points). The total score for the eight questions is taken as the final value; the higher the score, the worse the functionality.

The number of visits and treatment durations were also recorded for intergroup comparisons. The number of visits only included the number of times the patient received treatment or guidance. This parameter was not evaluated if the patient only received follow-up examinations at the hospital. Treatment duration was measured in weeks, referring to the total time from baseline (when the patient needed to visit the hospital for treatment or guidance) and excluding the time spent performing physical exercises at home.

### Statistical analysis

The sample size was calculated using PASS version 15 (NCSS, Kaysville, Utah, USA). A non-inferiority study design was adopted in this study, and the improvement value regarding pain intensity while chewing most types of food was considered the primary measurement of interest. The investigators set the test level at α = 0.025 and the efficiency at 1 - β = 0.90. Clinical significance was set at VAS scores > 2; hence, the non-inferiority margin was set to 2. The minimum sample size was calculated as 23 in the combined treatment group and 23 in the physical therapy group.

Propensity scores were estimated using logistic regression, and the calliper value was set at 0.05. Propensity score calculations were performed with 1:1 nearest-neighbour matching according to the patient’s age, sex, disease course (calculated from the onset of pain symptoms), dental intervention history, tooth grinding, tooth clenching, unilateral chewing, VAS scores (while chewing most types of food), MMO, and JFLS scores.

SPSS statistical software (version 23.0, Armonk, NY, USA) was used for data management and statistical analysis (including the aforementioned propensity score calculations). Means ± standard deviations (x ® ± s) were used to describe continuous data, whereas proportional data were expressed using ratios or percentages. When comparing baseline data between groups, the Shapiro–Wilk normality test was conducted for each group of continuous data, independent sample t-tests were used for data with a normal distribution, and the Mann–Whitney U test was used for data with an abnormal distribution. Chi-square tests were performed on the proportional data.

In comparing the two evaluated groups, we compared differences in VAS scores, MMO, and JFLS scores at 2, 4, and 12 weeks of follow-up (as compared with the baseline value before treatment) and compared the improvement ratio in joint crepitus. Improvement values in VAS scores, MMO, and JFLS scores in each group were tested for normality. If these values conformed to a normal distribution, an independent sample t-test was used; Mann–Whitney U-tests were used for data with abnormal distribution. Chi-square tests were used to compare the improvement ratio in crepitus within the joint area. Intra-group comparisons were performed to assess improvement after 2, 4, and 12 weeks compared with the baseline data. After the Shapiro–Wilk normality test for continuous variables, the paired sample t-test or Wilcoxon-rank sum test was used. The McNemar test was performed on the proportional data. In this study, p < 0.05 was set as the threshold for statistical significance.

## Results

Initially, 213 patients were enrolled in this study. Thirty-nine patients were subsequently excluded owing to the complications or refusal to participate in follow-up in a timely manner. Therefore, 174 patients were ultimately included in this study (42 and 132 patients in the combined treatment and physical therapy groups, respectively). After conducting PSM, each group included 32 patients (Fig. 1). Differences in patient medical and demographic characteristics between groups were insignificant (Table 1). All 64 patients completed the entire follow-up process.

**Table 1:** Comparisons of medical and demographic features and baseline values of the evaluated outcome variables

	CT (n = 32)	PT (n = 32)	P-value
Age (years)	59.66 ± 7.31	60.13 ± 7.21	0.716^b^
Sex (female/male)	31/1	31/1	1^c^
Course (months)	5.69 ± 4.98	4.80 ± 4.39	0.268^b^
TMJ sides (treated/total)	36/64	40/64	0.472^c^
ID1 (DDWOR/treated)	30/36	35/40	0.606^c^
ID2 (DDWR/treated)	2/36	2/40	0.914^b^
Dental	1/32	2/32	0.554^c^
Grinding	2/32	2/32	1^c^
Clenching	5/32	5/32	1^c^
Chewing	7/32	5/32	0.522^c^
VAS max	6.88 ± 0.91	6.69 ± 1.15	0.442^b^
VAS resting	2.78 ± 2.41	1.96 ± 2.24	0.154^b^
VAS opening	5.59 ± 1.93	4.59 ± 2.17	0.065^b^
VAS chewing	5.72 ± 1.49	5.75 ± 1.93	0.792^b^
MMO (mm)	28.00 ± 7.57	25.50 ± 6.23	0.154^a^
TMJ crepitus	21/32	17/32	0.309^c^
JFLS score	32.47 ± 10.20	35.84 ± 11.38	0.216^a^

CT, combined treatment; ID, internal derangements; JFLS, Jaw Functional Limitation Scale; DDWOR, disc displacement without reduction; DDWR, disc displacement with reduction; MMO, maximum mouth opening; CT, combined treatment; PT, physical therapy; TMJ, temporomandibular joint; VAS, visual analog scale

^a^Independent sample t-test

^b^Mann–Whitney U test

^c^Chi-square test

### Pain Intensity

**Fig. 6:**
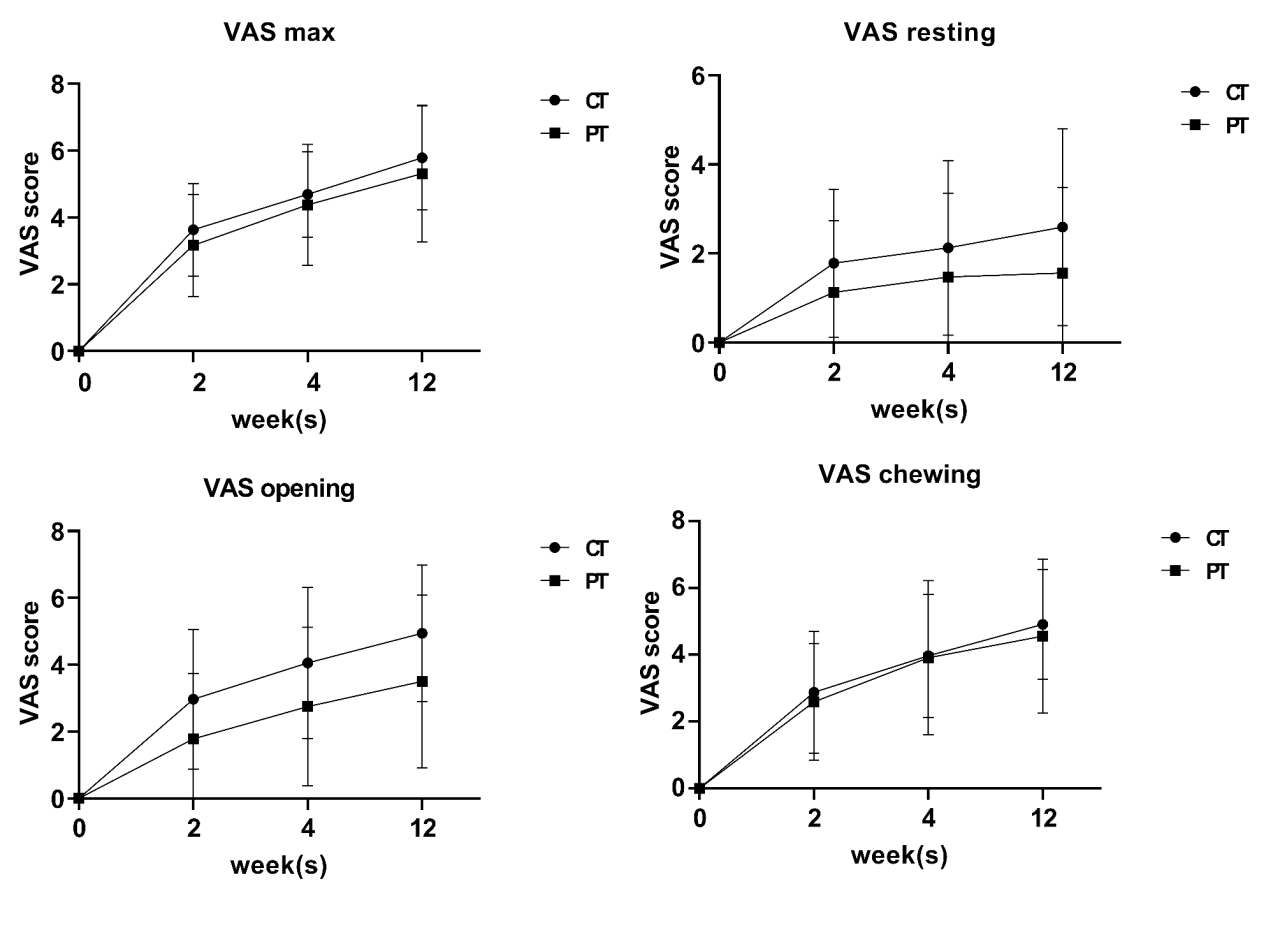
Improvement in pain intensity

Pain intensity when chewing most foods was the primary outcome of interest in this study. Before treatment, the average VAS scores for chewing pain in the combined treatment and physical therapy groups were 5.72 ± 1.49 and 5.75 ± 1.93, respectively, with no significant differences between groups (Table 1). Chewing pain improved significantly in the two groups at 2, 4, and 12 weeks of treatment. No significant difference was observed in improvement in chewing pain between the two groups at 2, 4, and 12 weeks (Fig. 6; Table 2).

**Table 2:** Improvement values and difference analysis at each observation time point

		Follow-up time		
Study variable	Groups	T1–T0	T2–T0	T3–T0
VAS max	CT	3.63 ± 1.39	4.69 ± 1.28	5.78 ± 1.56
	PT	3.16 ± 1.53	4.38 ± 1.81	5.31 ± 2.05
	P*-*value	0.347^b^	0.460^b^	0.429^b^
VAS resting	CT	1.78 ± 1.66	2.13 ± 1.96	2.59 ± 2.21
	PT	1.13 ± 1.60	1.47 ± 1.88	1.56 ± 1.92
	P*-*value	0.084^b^	0134^b^	0.051^b^
VAS opening	CT	2.97 ± 2.09	4.06 ± 2.26	4.94 ± 2.05
	PT	1.78 ± 1.96	2.75 ± 2.37	3.50 ± 2.58
	P*-*value	0.018^b,*^	0.045^b,*^	0.032^b,*^
VAS chewing	CT	2.88 ± 1.83	3.97 ± 1.84	4.91 ± 1.65
	PT	2.59 ± 1.74	3.91 ± 2.31	4.56 ± 2.31
	P*-*value	0.544^b^	0.753^b^	0.541^b^
MMO (mm)	CT	5.13 ± 4.23	7.78 ± 5.18	10.09 ± 5.74
	PT	6.81 ± 3.78	9.88 ± 4.47	11.50 ± 5.34
	P*-*value	0.034^b,*^	0.056^b^	0.309^b^
TMJ crepitus	CT	2/32	6/32	9/32
	PT	4/32	6/32	7/32
	P*-*value	0.581^c^	0.885^c^	0.357^c^
JFLS score	CT	17.47 ± 8.77	23.28 ± 9.96	27.53 ± 9.92
	PT	15.41 ± 9.93	20.81 ± 10.59	27.44 ± 12.16
	P*-*value	0.382^a^	0.364^a^	0.973^a^

CT, combined treatment; PT, physical therapy; JFLS, Jaw Functional Limitation Scale; MMO, maximum mouth opening; TMJ, temporomandibular joint; VAS, visual analog scale

T0, at baseline; T1, 2 weeks after the start of treatment; T2, 4 weeks after the start of treatment; T3, 12 weeks after the start of treatment

^*^Statistically significant

^a^Independent sample t-test

^b^Mann–Whitney U test

^c^Chi-square test

This study compared pain intensity in the two groups at rest and at the time of active mouth opening to the maximum capacity. Before treatment, no significant difference was observed in resting and mouth opening pain between the two groups (Table 1). However, the resting and mouth opening pain of the two groups improved significantly at 2, 4, and 12 weeks after the treatment commenced. In comparing the two groups, the improvement value of pain associated with mouth opening in the combined treatment group was better than that in the physical treatment group at 2, 4, and 12 weeks (p < 0.05). In contrast, no significant difference was observed in the improvement value regarding resting pain at the three observation points (Fig. 6; Table 2).

We recorded the maximum pain intensity felt by the two groups in the past week at each observation point. The maximum pain intensity improvement value in the two groups increased with time, with no significant difference between the two groups (Fig. 6; Table 2).

## MMO

**Fig. 7:**
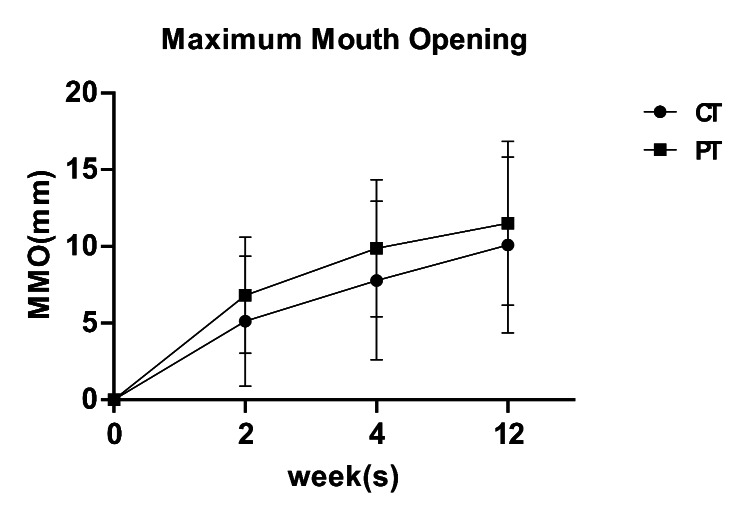
Improvement in maximum mouth opening

Before treatment, the mean values for MMO in the combined treatment and physical therapy groups were 28.00 ± 7.57 mm and 25.50 ± 6.23 mm, respectively, with no significant difference between the two groups (Table 1). MMO in the two groups improved significantly at 2, 4, and 12 weeks after the treatment commenced. When comparing the improvement value in MMO between the groups, the improvement value in MMO in the physical treatment group at 2 weeks was better than that in the combined treatment group (p < 0.05); however, no significant difference was observed between the two groups at 4 and 12 weeks (Fig. 7; Table 2).

### Joint Crepitus

At baseline, the proportions of joint crepitus determined by the physician at palpation in the combined treatment and physical therapy groups were 21/32 and 17/32, respectively, and the difference was insignificant (Table 1). On intra-group comparison, the crepitus ratio in the combined treatment group had improved significantly compared with the baseline only at 12 weeks (p < 0.05). No significant improvement was observed in the physical treatment group at the three observation points compared with the baseline. Disappearance in the joint crepitus improved compared with baseline, though we reported continued joint crepitus or new crepitus that had not improved. When comparing groups, no significant differences were observed in the improvement ratio for joint crepitus at 2, 4, or 12 weeks (Table 2).

### Jaw function (JFLS score)

At baseline, the mean JFLS scores in the combined treatment and physical therapy groups were 32.47 ± 10.20 and 35.84 ± 11.38, respectively, with no significant differences between groups (Table 1). The JFLS scores in the two groups improved significantly at 2, 4, and 12 weeks. When comparing the improvement values in JFLS scores between groups, no significant differences were observed at 2, 4, or 12 weeks (Table 2).

### Number of visits and treatment duration

The average number of visits in the combined treatment group was three (range, 3–4), whereas that in the physical treatment group was eight (3–23) (p < 0.05). The average treatment duration in the combined treatment group was 7 weeks (6–8), while that in the physical treatment group was 4 weeks (2–9) (p < 0.05). However, the physical therapy group required more treatment sessions, but the treatment duration was shorter.

### Complications and adverse reactions

No treatment or exercise-related complications were observed in this study. One patient had increased pain on the first day after the dexamethasone injection, which improved the following day rapidly. Two patients had increased joint pain after the administration of HA injection (which improved within 3 days). No adverse reactions related to exercise or physical therapy were observed.

## Discussion

A new combined treatment approach for DJD was adopted in this study. On the premise of rapid pain relief through injection therapy, professional physical therapists guided patients in performing home physical exercises to restore jaw function quickly. The control group used the standard physical therapy for DJD. Our results revealed that pain intensity, MMO, and JFLS scores of patients in the combined treatment and physical therapy groups improved at 2, 4, and 12 weeks, and the degree of improvement increased with time. The crepitus ratio improved significantly only in the combined treatment group at 12 weeks. Compared with the physical therapy group, improvements in pain associated with mouth opening in the combined treatment group were greater after 2, 4, and 12 weeks. The improvement in MMO in the physical therapy group was better than that in the combined treatment group at 2 weeks. The number of visits in the combined treatment group was significantly lower than that in the physical therapy group, while the treatment duration was longer.

Injection therapy is one of the most common treatment methods for DJD. Corticosteroid injections can quickly reduce joint pain and eliminate joint effusion. However, this treatment modality carries a risk of articular cartilage damage. Therefore, corticosteroids should not be administered more than four times yearly [36, 37]. HA injection for DJD involves injecting HA directly into the articular cavity to maintain an appropriate concentration of low molecular weight solution, improve rheological properties, and reduce friction force in the articular cavity to improve symptomology [38, 39]. In recent years, intra-articular injection of HA in the TMJ has proven effective in alleviating DJD pain symptoms [40] and promoting condylar bone repair [41].

Physical therapy, one of the initial treatment options for DJD, includes various measures. The treatment modalities such as the use of low-level ultrasound, interference currents, low-intensity lasers, and ultrashort waves can reduce inflammation, promote microcirculation, and relax masticatory muscles to obtain analgesic effects and improve joint mobility [31–33, 42]. Manual therapy can improve blood circulation, reduce muscle spasms, relax muscles around joints, rearrange soft tissue, reduce joint adhesion, increase the joint’s range of motion, and reduce pain [43]. Manual therapy combined with exercise training can reduce symptoms and increase the range of motion in patients with TMD [44, 45].

Physical exercise is one of the commonly used treatments for musculoskeletal pain and related diseases, including TMD. Physical exercise is the basis of rehabilitation for musculoskeletal diseases [46]. Physical exercise can restore normal functionality by changing sensory input; reducing inflammation, pain, and muscular activity; improving muscle coordination and strength; and promoting tissue repair and regeneration [47]. Emerging evidence emphasizes that chronic musculoskeletal pain disease treatments, including exercise training for the surrounding musculoskeletal system and changes in cortical neuroplasticity caused by chronic pain, provide the greatest potential for successful rehabilitation [48]. Physical exercise can be used to treat painful TMD. Previous studies on using exercise to treat painful TMD have revealed that exercise therapy positively impacts various clinical symptoms of painful TMD with low invasiveness and high economic effectiveness [29].

Previous studies on patients with TMD have revealed that exercise training alone or combined with other treatment methods can improve patients’ mouth-opening capabilities and mandibular movement range and reduce pain intensity [49, 50]. A previous study of older patients with TMJ osteoarthritis revealed that a home-based exercise program significantly improved pain, functionality, and joint disc position [51]. Previous studies have proven that exercise training can improve patients’ pain levels and their mouth-opening and mandibular functionality, which is consistent with the conclusions of our study.

More specifically, this study discovered that the improvement in pain associated with mouth opening in the combined treatment group was better than that in the physical treatment group at 2, 4, and 12 weeks of follow-up, which could be attributed to the benefits of dexamethasone and HA injection and was also related to the high frequency and longer duration of exercise in the combined treatment group. Similarly, improvement in mouth opening in the physical therapy group was better than that in the combined treatment group after 2 weeks. We believe that this finding is related to the early manual therapy received by the physical therapy group; however, the difference in the MMO improvement value (< 2 mm) may not have clinical significance.

In addition, the number of visits required for the combination therapy group is substantially lower than that required for physical therapy (the latter has higher time and economic benefits for patients). The treatment duration in the physical therapy group was significantly shorter, indicating that physical therapy conducted two to three times a week is conducive to rapid recovery.

No treatment complications or adverse reactions were observed in the physical therapy group. In the combined treatment group, three patients had increased pain after injection that improved within 1–3 days. In conclusion, both treatment regimens are safe for older patients with DJD.

The following are the strengths of our study: (1) all participants were older individuals with DJD, ensuring a homogenous sample; (2) a new treatment protocol, that is, injection therapy combined with home physical exercise, was studied and introduced; and (3) in addition to evaluating the conventional symptom indicators, the treatment durations were compared to evaluate the efficiency and benefits of the treatment methods.

This study had some limitations. First, this was a prospective cohort study, and the level of evidence was lower than that obtained using randomized controlled study designs. Second, no follow-up imaging changes were evaluated. Third, different drugs were used according to each patient’s conditions in the injection therapy group. Lastly, the treatment components in the two groups included physical exercise. More specifically, although the two groups differed in frequency and treatment completion (at home compared with that at hospital with therapist assistance), the treatment methods of the compared interventions overlapped. In addition, whether injection therapy is combined with family exercise training or physical therapy, the evaluated interventions comprised different treatments, and the effectiveness of a single method cannot be distinguished. We also observed that previous studies on physical exercise mostly focused on patients with TMD but less on DJD classification, making our report less comparable with prior research.

## Conclusion

This study aimed to explore the efficacy of injection therapy combined with home physical exercise compared to physical therapy alone. Our results revealed that injection therapy combined with home physical exercise treatment and physical therapy improved the pain intensity, MMO, joint crepitus, and mandibular function of older patients with DJD significantly. Combined treatment improved pain associated with mouth opening within 12 weeks, and the number of required visits was less than that for physical therapy. Physical therapy improved patients’ mouth-opening capabilities in a short time (2 weeks), and the treatment duration was shorter (which is more conducive to a more expeditious recovery).

This study has certain guiding significance for the treatment selection of older patients with DJD. Physical therapy is advantageous because it is non-invasive and can effectively control the symptoms of DJD quickly. The combination treatment has advantages of requiring fewer visits and better control of pain associated with mouth opening. Therefore, injection therapy combined with family physical exercise is a good choice if the older patient complains of pain associated with mouth opening or desires fewer visits.

## Data Availability

The datasets used and/or analysed during the current study are available from the corresponding authors on reasonable request.

## Declarations

### Guidelines

All study procedures were performed in full compliance of the principles of the Declaration of Helsinki. The reporting of this work followed the guidelines delineated in the STROBE statement.

## List of abbreviations

DJD: degenerative joint disease
HA: hyaluronic acid
JFLS: jaw functional limitation scale
MMO: maximum mouth opening
MRI: magnetic resonance imaging
PSM: Propensity score matching
TMD: Temporomandibular disorder
TMJ: temporomandibular joint
VAS: visual analogue scale
